# TmAbd5 Is Essential for Endocuticle Formation in the Yellow Mealworm, *Tenebrio molitor*

**DOI:** 10.3390/insects17060601

**Published:** 2026-06-08

**Authors:** Rongrong Yu, Haoran Wang, Gaohua Liu, Xiaoming Zhao, Mureed Abbas, Nan Chang, Xuekai Shi, Yujing Yang, Yuping Zhang

**Affiliations:** 1 College of Biological Sciences and Technology, Taiyuan Normal University, Jinzhong 030619, China; yurrong@163.com (R.Y.); 13796619712@163.com (H.W.); liu426482@163.com (G.L.); 15296778753@163.com (N.C.); xuekaishi@163.com (X.S.); yyjing@nwafu.edu.cn (Y.Y.); 2 Shanxi Key Laboratory of Nucleic Acid Biopesticides, Institute of Applied Biology, Shanxi University, Taiyuan 030006, China; zxming@sxu.edu.cn (X.Z.); malikmureed05@gmail.com (M.A.)

**Keywords:** *Tenebrio molitor*, TmAbd5, RNA interference, biological function, endocuticle

## Abstract

*Tenebrio molitor* (Coleoptera: Tenebrionidae) is a suitable candidate for use as a biomass resource, recognized for its large-scale breeding and the high nutritional value of its protein content. Feeding obese Zucker rats the cuticle of *T. molitor* enhances fatty liver metabolism through the mediation of gut microorganisms. Cuticular proteins are demonstrated to be pivotal in the formation of the insect cuticle throughout the developmental stage. The endocuticle structural glycoprotein (Abd) belongs to the RR-1 subclass, a major group of structural cuticular proteins characterized by the conserved Rebers–Riddiford (RR) motif. Nevertheless, there remains a paucity of research into the molecular properties and functions of SgAbd (endocuticle structural glycoprotein) in Coleoptera. In this study, we successfully identified and described the gene *TmAbd5* in *T. molitor*. The coding sequence of *TmAbd5* is 306 bp, corresponding to a 101-aa protein. The functional domain predicted that TmAbd5 consists of a signal peptide and a chitin-binding domain 4 (ChtBD4). Motif prediction analysis indicated that TmAbd5 belongs to the CPR (cuticular proteins with Rebers–Riddiford consensus) family with the RR-1 motif. Expression analysis revealed that *TmAbd5* is upregulated in the integument, particularly during the first three days of development in the 13th instar stage. Although the RNAi-mediated silencing of *TmAbd5* did not cause apparent phenotypic abnormalities and the insects successfully molted into pupae, histological examination revealed a substantial thickening of the endocuticle at 72 h post-pupation, along with a notable increase in lamellar spacing and a disrupted pore canal. In summary, TmAbd5 contributes to the formation and structural organization of the endocuticle, which provides a theoretical basis for the screening of target genes for cuticle development and for the effective utilization of cuticle resources in *T. molitor*.

## Simple Summary

The yellow mealworm, *Tenebrio molitor*, is an important edible insect widely bred for its efficient growth and high-quality protein. Its outer covering, called the cuticle, plays a vital role in growth and protection. The cuticle contains special proteins that help form and strengthen this protective layer during development. In this study, we identified and examined a specific cuticle protein named TmAbd5 in the yellow mealworm to better understand its role. We found that this protein is mainly produced in the body surface and is especially active during the first three days of the late developmental larvae stage. When the production of this protein was reduced, the insects were still able to molt normally. However, important structural changes were observed in the inner layer of the cuticle, which became thicker and exhibited wider spacing between layers after pupation. These findings improve our understanding of how the insect’s outer covering forms and develops. This knowledge may support future efforts to utilize mealworms, including improving their breeding and making more effective use of their biological resources.

## 1. Introduction

The insect integument consists of a secreted multi-layered cuticular structure and single-layered epidermal cells. The cuticle comprises three horizontal layers: the envelope, epicuticle, and procuticle [1,2]. The envelope, a complex structure composed of lipids and proteins, provides protection from dehydration and pathogens. The epicuticle, primarily made of lipids and proteins, helps maintain body shape and rigidity. The procuticle is a helicoidally arranged structure consisting of chitin fibrils and cuticular proteins (CPs) assembled into a protein–chitin matrix, providing the exoskeleton with flexibility, strength, and stability [3]. To adapt growth and avoid the restriction of a rigid exoskeleton, insects periodically undergo ecdysis, during which the old cuticle is degraded and replaced by a newly synthesized one [4,5,6]. The exocuticle and epicuticle are deposited before apolysis, whereas the endocuticle is formed after ecdysis and continues to develop until the next apolysis [7].

To date, a large number of cuticular proteins have been discovered in various insects, including *Manduca sexta*, *Nilaparvata lugens*, and *Anopheles gambiae* [8,9,10]. Cuticular proteins are divided into CPAPs (cuticular proteins analogous to peritrophins), CPFs (cuticular proteins with forty-four highly conserved amino acids), CPFLs (CPFs like conserved amino acids in the C-terminal), CPLCs (cuticular proteins of low complexity), CPGs (cuticular proteins rich in glycine), CPRs, and CPHs (cuticular proteins hypothetical), among others, according to the conserved motifs. Among these, the CPR family, as the most prominent cuticular protein family, includes three subclasses: RR-1, RR-2, and RR-3 [11]. Cuticle proteins are essential for cuticle formation throughout insect development; a disruption to or reduction in their expression can severely affect cuticle morphology, integrity, and function, often resulting in lethality. For example, in *Aedes aegypti*, silencing *AaCPR100A* disrupted endocuticle formation in larvae, significantly reduced the egg hatching rate, and increased mortality [12]. In *Spodoptera exigua*, knockout of the cuticular protein gene *SeCPG316* resulted in a blackish and hardened cuticle, abnormal pupation, and increased mortality [13]. In *N. lugens*, silencing fifteen cuticle protein genes from the CPR family resulted in insect mortality [10].

The abdomen serves as the central site for digestion and reproductive metabolism in insects. Multiple *Abd* genes have been identified in the abdominal cuticle across various insect orders. In Orthoptera, eight *Abd* family genes have been identified in *Schistocerca gregaria* and *Locusta migratoria* [14,15]. In *L. migratoria*, LmAbd5 plays a key role in the formation of the endocuticle structure [16]. In Lepidoptera, Abd5 from *Helicoverpa armigera* contributes to cuticle compaction and lamellar organization, thereby enhancing resistance to fenvalerate [17]. In Hemiptera, AgAbd2 is required for maintaining endocuticle thickness during the molting of *Aphis gossypii*, and its transcript levels are significantly upregulated in response to *Metarhizium anisopliae* infection, providing protection against fungal invasion [18,19]. In Coleoptera, TcZelda (Zinc finger protein) and TcAbd cooperate to regulate embryonic and wing development, and the silencing of *TcZelda* results in the significant downregulation of *Abd* gene expression; however, the molecular signatures and physiological functions of *Abd* genes in this order remain largely unexplored [20].

The yellow mealworm, *Tenebrio molitor* (Coleoptera: Tenebrionidae), is an essential edible insect species that provides efficient biomass instead of food and feed, with large-scale propagation and high nutritional value [21,22,23,24]. Mammals reared using the cuticle of *T. molitor* as feed exhibit enhanced fatty liver metabolism through the mediation of gut microorganisms [25]. In this study, we identified five endodermal structural protein genes (*TmAbd2*, *TmAbd4*, *TmAbd5*, *TmAbd8* and *TmAbd9*) from the transcriptome of *T*. *molitor*. Furthermore, we selected *TmAbd5* to explore its molecular characteristics and biological function. The expression profile of *TmAbd5* indicated that it has a high transcription level in the integument, particularly during the first three days of the 13th instar larval stage. The RNA interference-mediated knockdown of *TmAbd5* led to significant thickening of the endocuticle at 72 h post-pupation, accompanied by an increased interval between the lamellae and disrupted pore canal within the endocuticle structure. These findings elucidate the molecular mechanism of cuticle formation during development in *T. molitor*, and the results enrich our knowledge of insect cuticular proteins, thus providing an essential theoretical basis for screening target genes for cuticle development and the further effective utilization of cuticle resources in *T. molitor* [26].

## 2. Materials and Methods

### 2.1. Insect Rearing

The *T. molitor* individuals were purchased from the Xinyang Mixue Industry Co., Ltd. (Luoyang, China), and reared in an artificial climate incubator (Boxun, Shanghai, China) under controlled conditions: a temperature of 26 °C, relative humidity of 70%, and a light/dark cycle of 18:6. Larvae were fed with fresh wheat bran and provided with lettuce to maintain adequate body moisture.

### 2.2. Prediction and Analysis of the Structural Features of TmAbd5

The transcript sequence of *TmAbd5* was retrieved from the *T. molitor* transcriptome database. Based on the cDNA sequence, the translated protein sequence, molecular mass, and isoelectric point were predicted according to the Expasy translate and Expasy Compute pI/Mw tools (https://www.expasy.org/). The conserved domain was identified using the SMART online website(http://smart.embl-heidelberg.de/), and SignaIp 4.1 was employed for signal peptide prediction. Conserved protein motifs were represented with the WebLogo website (https://weblogo.threeplusone.com/create.cgi). The Abd5 homolog amino acids were clustered and a phylogenetic tree was built in MEGA.11 with 1000 bootstrap replicates. The homology sequence of Abd in the different insects for construction of the phylogenetic tree is shown in Table 1.

### 2.3. Expression Pattern of TmAbd5 in Different Stages, Tissues and Developmental Days

For the developmental stage expression analysis, four biological replicates were prepared, each consisting of ten eggs and three individuals from each developmental stage, including early instar larvae, middle instar larvae, late instar larvae, pupae, and adults. For the tissue expression pattern, the integument, fat body, Malpighian tubules, foregut, midgut, and hindgut were dissected and collected from forty 2-day-old 13th instars. For developmental expression profiling in the integument, samples were collected from 180 individuals across different developmental days. Each assay included four biological replicates and two technical replicates, each consisting of three larvae. RNA concentration was detected using a NanoDrop 2000 spectrometer (Thermo Fisher, Waltham, MA, USA), and integrity was confirmed using 1% agarose gel (Sangon, Shanghai, China). Reverse transcription was carried out using HiScript III RT SuperMix (Vazyme, Nanjing, China). Using Primer 5.0, specific primers were designed and subsequently synthesized by Sangon Biotech (Table 2). Then, 20 µL of reaction mixture was prepared for real-time quantitative PCR (RT-qPCR), including 10 μL of SYBR Premix^Ex^Taq^TM^II (TOYOBO, Tokyo, Japan), 6.4 μL of ddH_2_O (sterile deionized water), 2 µL of cDNA reverse transcription template, and 0.8 µL (10 µM) each of forward and reverse primers. The RT-qPCR assay was performed on a Step-One-Plus Real-Time PCR System (Applied Biosystems, Waltham, MA, USA) with a reaction procedure consisting of pre-denaturation at 95 °C for 10 s, followed by 40 cycles of denaturation at 95 °C for 5 s, and annealing and elongation at 60 °C for 30 s. Then, a melting curve was created from the detection of the fluorescence intensity of the samples from 60 °C to 95 °C, which determined the specific gene-specific peaks and avoided primer dimers. The transcription levels of *TmAbd5* were quantified using the 2^−ΔΔCt^ method. Discrepancies between samples were calibrated using *RPS3* as an internal reference.

### 2.4. Double-Stranded RNA (dsRNA) Preparation and Silence Efficiency Detection

A pair of dsRNA primers with the T7 promoter sequences shown in Table 2 was designed using Primer 5.0 software and synthesized by Sangon Biotech (Sangon, Shanghai, China). A 25 μL amplification reaction system was used, containing 12.5 μL PCR MasterMix (Tiangen, Beijing, China), 0.5 μL each of forward and reverse primers, and 1 μL cDNA of *TmAbd5* template and sterile deionized water (Sangon, Shanghai, China) up to the final volume. The PCR conditions were 95 °C pre-denaturation for 1 min; 35 cycles of 95 °C denaturation for 30 s, 60 °C annealing for 30 s, and 72 °C extension for 1 min; and a final incubation at 72 °C for 8 min. PCR products were purified using a gel recovery kit (Vazyme, Nanjing, China), and dsRNA was synthesized using the T7 RNAi Express kit (Promega, Madison, WI, USA). ds*GFP* and ds*TmAbd5* were detected using configured 1% agarose gel electrophoresis.

An amount of 1 μL of ds*TmAbd5* (0.5 μg) was administered by piercing the intersegmental membrane of the 3rd and 4th abdominal segments of 2-day-old 13th instars with a microinjector (Gaoge, Shanghai, China). The RNAi control group was injected with dsRNA specific to green fluorescent protein (ds*GFP*). Integument samples were dissected from the larvae overnight and ds*TmAbd5* was obtained for silencing efficiency analysis via RT-qPCR, with four biological replicates (each containing three larvae) and two technical replicates.

### 2.5. Phenotypic Observation

The equivalent dsRNA was injected into the thirty 2-day-old 13th instars according to the description in Section 2.4. Subsequently, the larvae were incubated and phenotypic changes in *T. molitor* were observed every day until the larvae molted into adults. The phenotypes of the adults derived from ds*GFP*- and ds*TmAbd5*-injected larvae were recorded and analyzed.

### 2.6. Microstructure Observation with H&E Staining

Six 2-day-old 13th instar were collected for the injection of equivalent ds*GFP* or ds*TmAbd5* according to the description in Section 2.4. The integuments of the second abdominal segment were dissected after molting into the pupal stage at 72 h and fixed with 3% glutaraldehyde at 4 °C. The samples were then embedded, sectioned, and dewaxed. Hematoxylin and eosin (H&E) was applied as previously described [27]. After dehydration and sealing, nine images of each section were captured under a DS-Ri2 inverted fluorescence microscope (Nikon Corp., Tokyo, Japan). The assay was carried out with three biological replicates, with each containing one larva.

### 2.7. Ultrastructure Observation by TEM

A transmission electron microscope (TEM) (HITACHI, Tokyo, Japan) was used to determine the consequences of *TmAbd5* silencing in the ultrastructure of the integument. After silencing the transcription of *TmAbd5* according to the description in Section 2.4, six integuments from the third abdominal segment were dissected from larvae 72 h after molting into the pupal stage, cut into small pieces (approximately 2 mm^3^), and then fixed, dehydrated, impregnated, and embedded. The sections were stained with uranyl acetate (SPI Supplies, West Chester, PA, USA) and 2.6% lead nitrate (Sigma-Aldrich, Louis, MO, USA) solution according to established protocols [28]. Twelve images were captured for each sample with an LSM 880 confocal laser scanning microscope (Carl Zeiss, Oberkochen, Germany). The assay was carried out with three biological replicates, each containing one larva.

### 2.8. Statistics

The expression characteristics of *TmAbd5* in various stages, tissues and different developmental days of the integument were evaluated via one-way analysis of variance (ANOVA) with Tukey’s test using SPSS 22.0 software. Different letters on the histograms indicate significant differences. Student’s t test was employed for the comparison of silence efficiency and endocuticle thickness after injection with dsRNA. Asterisks on the column show the considerable difference between ds*GFP* and ds*TmAbd5* groups (** *p* < 0.01; *** *p* < 0.001).

## 3. Results

### 3.1. Bioinformatic Analysis of TmAbd5

**Figure 1:**
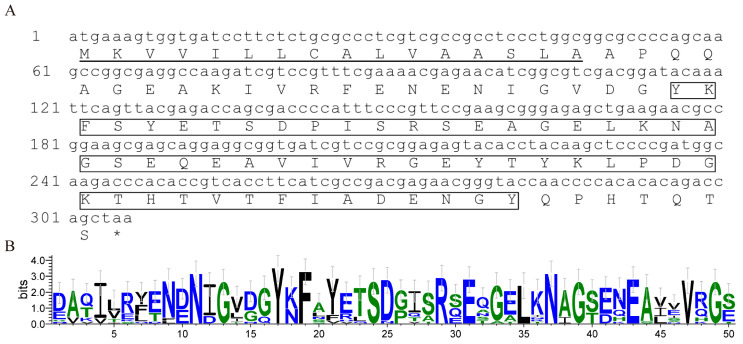
The full-length cDNA sequence and conserved motif prediction of TmAbd5. (**A**) The nucleotide, predicted amino acid, and conserved function prediction. The signal peptide is underlined, and the chitin-binding domain (ChtBD4) is shown in the box. (**B**) The conserved motif analysis of TmAbd5 was carried out using Weblogo website. Asterisk showed the Stop codon.

In accordance with the *T. molitor* transcriptome database, a query was generated using the keywords “endocuticle structural glycoprotein”. Consequently, the full-length *TmAbd5* sequence was retrieved, which consisted of the 306 bp coding region of *TmAbd5* and the 101 amino acids of the encoding protein. Conserved functional domain analysis showed that TmAbd5 contains a signal peptide (1–16 aa) and a chitin-binding domain type 4 (ChtBD4; 38–93 aa) (Figure 1A). The predicted isoelectric point (PI) and molecular weight (MW) of the protein are 4.90 and 10.97 kDa, respectively. Motif analysis revealed that TmAbd5 possesses the RR consensus (Figure 1B). The accession number of TmAbd5, which we submitted to the GenBank database, is XP068904910.1.

### 3.2. Phylogenetic Analysis of TmAbd5

**Figure 2:**
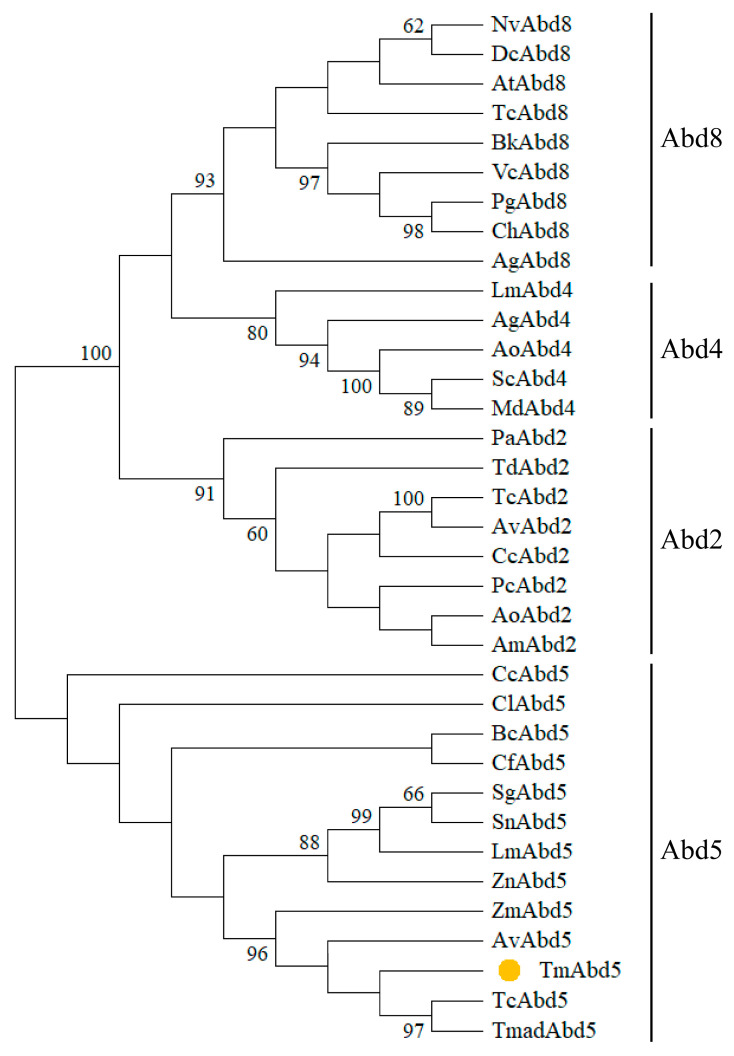
A phylogenetic tree of the Abd5 homologous sequence in different insects. The phylogenetic tree was constructed with 1000 independent analyses by MEGA.11 software. The TmAbd5 is marked with a solid orange circle. Number on the branch showed the confidence level.

The phylogenetic analysis showed that TmAbd5 from *T. molitor* clusters with Coleoptera Abd5 homologs. Specifically, TmAbd5 exhibits the closest evolutionary relationship with Abd5 from *Tribolium castaneum* and *Tribolium madens*, while it is most distantly related to Abd5 of *Asbolus verrucosus* (Figure 2).

### 3.3. Expression Traits of TmAbd5 in Various Stages, Tissues and Different Developmental Days of the Integument

**Figure 3:**
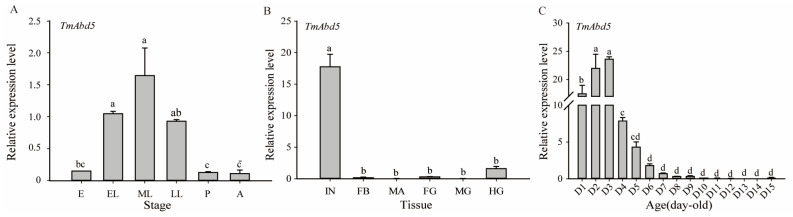
The specific expression pattern of *TmAbd5* in the different stages, tissues and developmental days. (**A**) Relative expression levels of *TmAbd5* across different developmental stages. E, eggs; EL, early instar larvae; ML, middle instar larvae; LL, late instar larvae; P, pupae; A, adults. (**B**) The relative expression of *TmAbd5* among tissues. IN: integument; FB: fat body; MA: Malpighian tubules; FG: foregut; MG: midgut; HG: hindgut. (**C**) The relative expression of *TmAbd5* at different developmental days. Data are represented in the form of means ± SE. Different letters on the bars indicate significant differences (*p* < 0.05, one-way ANOVA and Tukey analysis). *RPS3* was used as a reference gene to calibrate the transcription level of *TmAbd5*. Each assay contains four biological replications.

The expression characteristics of *TmAbd5* in various stages, tissues and at various developmental days were assessed. The developmental stage expression analysis revealed that *TmAbd5* is highly expressed during the larval stages compared with the egg, pupal, and adult stages (Figure 3A). Therefore, the larval stage was selected for subsequent tissue-specific expression profiling. The relative transcription level of *TmAbd5* in various tissues indicated that it was more highly expressed in the integument compared with other tissues (Figure 3B). To further examine its expression during development, the transcript levels of *TmAbd5* in the integument were assessed across different developmental days of the 13th instar larval stage. The results demonstrated that *TmAbd5* expression was significantly higher during the first three days, after which it gradually declined from day 4 to day 15 (Figure 3C).

### 3.4. TmAbd5 Is Irreplaceable in Endocuticle Formation

**Figure 4:**
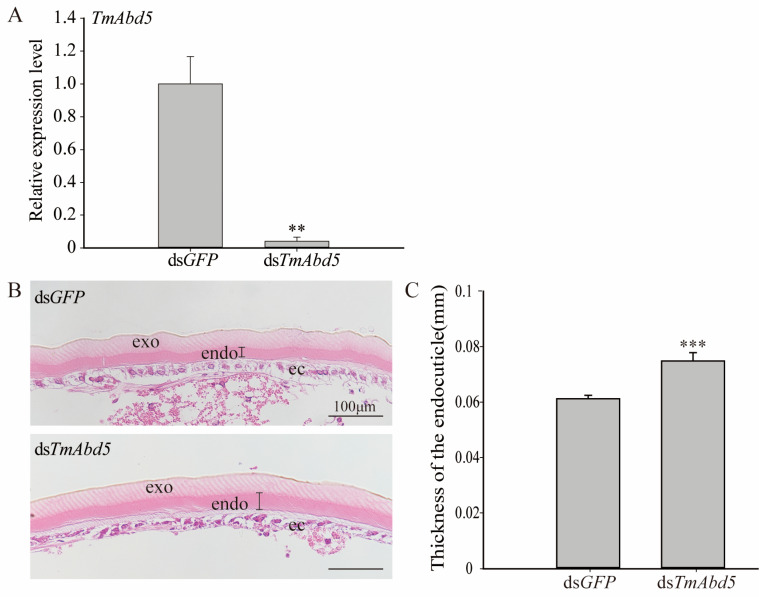
Silence efficiency and microstructure after ds*TmAbd5* injection. (**A**) The relative expression of *TmAbd5* after dsRNA injection. Asterisk on the bar indicates significant difference after injection of ds*GFP* (control) and ds*TmAbd5*. Statistically significant difference was analyzed with Student’s *t* test (** *p* < 0.01). (**B**) Microstructure observation of cuticle after injection of ds*GFP* and ds*TmAbd5*. Exo: exocuticle, endo: endocuticle. Scale bar is 100 μm. (**C**) Comparison of endocuticle thickness after injection of ds*GFP* and ds*TmAbd5* at 2 days old with H&E staining. Data are represented in the form of means ± SE. Statistically significant difference was analyzed using Student’s *t* test (*** *p* < 0.001).

RNA interference (RNAi) was employed to silence the transcription level of *TmAbd5*, followed by phenotypic observation. Silencing efficiency assays indicated that the transcript level of *TmAbd5* was obviously reduced by 91.7% at 24 h after the injection of ds*TmAbd5* relative to controls (Figure 4). Subsequent histological examination using H&E dyeing revealed that the endocuticle of the ds*TmAbd5*-treated group became notably thicker than that of the control group with an increase of 22.4% (Figure 4B,C), which indicated that TmAbd5 is involved in endocuticle formation.

### 3.5. TmAbd5 Influence on the Compactness of the Endocuticle

**Figure 5:**
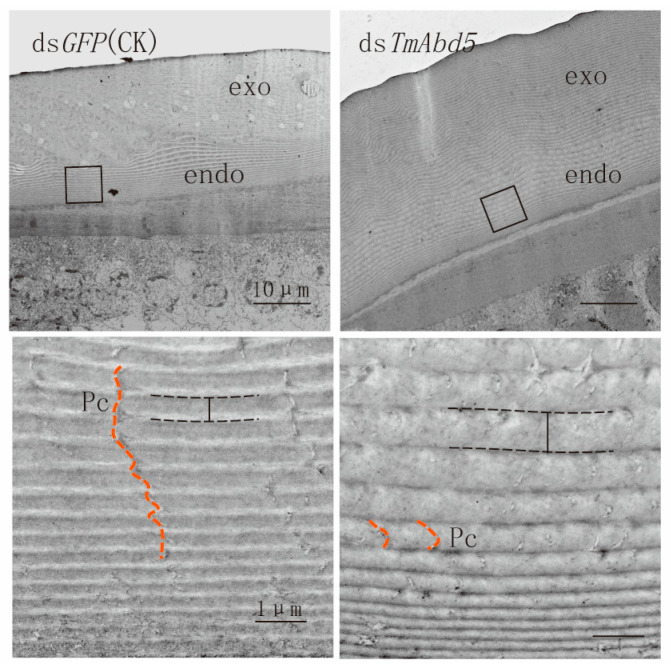
The ultrastructure of the cuticle was observed using TEM after the injection of ds*GFP* and ds*TmAbd5*. Exo: exocuticle, endo: endocuticle, Pc: pore canal. Scale bars are 10 μm and 1 μm. Box indicates the magnifying area of the endocuticle. The black line shows the cuticle lamella. Black dashed lines indicate the boundary of the lamella, and the orange dashed line shows the pore canal going through the lamellae.

To observe whether ds*TmAbd* had an effect on lamellar organization, the ultrastructure of the cuticle was observed after the injection of ds*TmAbd5* using TEM. The result revealed that, following the injection of ds*GFP*, the endocuticle is tight and the pore canal is long and continuous across the lamellar. However, the lamellar structure of the endocuticle in the ds*TmAbd5* group appeared wider and looser, and the pore canal was interrupted and discontinuous (Figure 5). Together, these findings demonstrate that *TmAbd5* is indispensable for lamellar organization and pore canal formation in the endocuticle of *T. molitor*.

## 4. Discussion

The insect cuticle, which performs essential physiological functions, consists of four distinct layers: the envelope, epicuticle, exocuticle, and endocuticle [29]. Cuticular proteins are the predominant structural proteins in the cuticle. The CPR family is known to be the most prominent and is classified into three clusters: the RR-1 subfamily, predominantly expressed in the soft cuticle; the RR-2 subfamily, mainly expressed in the rigid cuticle; and the RR-3 subfamily, which remains relatively less studied at present [11]. In this study, a post-molting cuticle protein encoded by *TmAbd5* was retrieved from the transcriptome database of *T. molitor*. The functional domain prediction of TmAbd5 revealed that it consists of a signal peptide and a chitin-binding domain (ChtBD4), indicating that TmAbd5 is a secretory protein with chitin-binding capability. Conserved motif analysis further confirmed that TmAbd5 contains a typical RR-1 conserved motif, which infers that it belongs to the CPR family. These results are consistent with previous findings on the LmAbd5 protein in *L. migratoria* [16]. Moreover, Abd5 homologs from Coleoptera species formed a distinct clade with high bootstrap support, suggesting a close evolutionary relationship among Abd5 proteins within this order.

The expression profile of *Abd* genes highlighted tissue- and stage-specific patterns across different developmental periods. For example, in *L. migratoria*, *LmAbd1*, *LmAbd2*, *LmAbd6*, and *LmAbd9* are highly expressed in the cuticle, whereas *LmAbd5* shows high expression in the foregut, hindgut, and cuticle [15,16,30,31]. In *Reticulitermes aculabialis*, *Abd2* and *Abd9* exhibited high expression levels in the head of worker ants, with significantly higher expression levels in nymphs than in adults [32]. In *Aphis citricola*, *Abd2* is upregulated in the abdominal region [18]. In the study, we found that *TmAbd5* inhibited a high expression in the integument, which is similar to the cuticle’s high expression pattern in *H. armigera* [17], suggesting a potential role in cuticle formation. Furthermore, the analysis of *TmAbd5* expression in the integument during the 13th instar larval stage showed that transcript levels were highest during the first three days after molting. In *L. migratoria*, *LmAbd5* is highly expressed 0–72 h after molting, which appears to be similar to the expression profile in the integument [16]. These findings indicate that cuticle formation is a dynamic developmental process, with endocuticle formation completed mainly within three days after molting [33]. The expression pattern of *TmAbd5* coincides with this critical period, suggesting that it is responsible for the formation and structural maintenance of the endocuticle in *T. molitor*.

Abd family proteins play crucial roles in the cuticle, as well as in growth and development. In *L. migratoria*, Abd family proteins are required for the maintenance of the cuticle inter-segmental membrane and the lamellar structure of the endocuticle, and for facilitating oviposition [15,16,29,30]. Similarly, in *Frankliniella occidentalis*, the Abd1 protein has been shown to be necessary for oviposition, longevity, and body shape maintenance [34]. Functional explorations of the Abd5 protein have demonstrated that it is responsible for the maintenance of the cuticle structure in *L. migratoria* and *H. armigera*. In *L. migratoria*, when *LmAbd5* is silenced, the nymphs are able to molt successfully despite the lack of a normal phenotype; furthermore, the lamellar of the endocuticle is looser and the endocuticle is thicker [16]. In *H. armigera*, the molt phenotype is not observed. However, the silencing of *HaAbd5* leads to the tight cuticle lamellar architecture becoming looser and thicker, resulting in the greater susceptibility of the cuticle to fenvalerate penetration [17]. In this study, RNAi combined with microscopic and ultrastructural observation was adopted to elucidate the actions of TmAbd5 in *T. molitor*. Although the larvae injected with ds*TmAbd5* successfully molted into the pupal stage without apparent phenotypic abnormalities, microscopic and ultrastructural analyses found that the endocuticle exhibited significantly increased interlamellar spacing, leading to a looser architecture and an overall thicker endocuticle. This is similar to the phenotype of *L. migratoria* and *H. armigera*, showing that the biological functions of Abd5 in insects were conserved. Furthermore, we found that the pore canal, which is responsible for transporting lipids to the envelope, was discontinuous and interrupted in the endocuticle, showing the specific biological function of *TmAbd5* in the cuticle. However, the penetration, sensitivity, and water retention of the cuticle should be explored further in future research. Overall, the results suggest that TmAbd5 is responsible for lamellar structure organization and pore canal formation, thus positioning it as a target gene for the development of *T. molitor*. In future work, we will combine the target gene and nanoparticles in oral RNAi to determine more applications [35].

## 5. Conclusions

In this study, the molecular properties and functions of TmAbd5 in *T. molitor* were established. The results demonstrated that TmAbd5 belongs to the CPR family, subclass RR-1. *TmAbd5* was highly expressed in the integument and contributed to the formation of the endocuticle of *T. molitor*. In summary, the results offer insights into the architecture and function of the cuticle during the growth and development of *T. molitor*. Moreover, this study enhances the current knowledge of insect cuticular proteins and offers an essential theoretical basis for developing and utilizing cuticular resources in *T. molitor*.

## Figures and Tables

**Table 1:** Homologous sequence of Abds used for phylogenetic tree development.

Orders	Species	Genes	Accession Numbers	Access Date
Siphonaptera	*Ctenocephalides felis*	*CfAbd5*	XP_026468045.1	1 October 2018
Diptera	*Bradysia coprophila*	*BcAbd5*	XP_037044862.153	5 March 2026
	*Condylostylus longicornis*	*ClAbd5*	XP_055373233.1	24 April 2023
	*Stomoxys calcitrans*	*ScAbd4*	XP_013106443.1	2 September 2023
	*Musca domestica*	*MdAbd4*	XP_005184506.2	24 August 2023
	*Anastrepha obliqua*	*AoAbd4*	XP_054739170.1	3 April 2023
	*Anopheles gambiae*	*AgAbd4*	XP_318989.4	19 December 2023
Neuroptera	*Chrysoperla carnea*	*CcAbd5*	XP_044736842.1	27 October 2021
	*Chrysoperla carnea*	*CcAbd2*	XP_044743078.1	27 October 2021
	*Phlebotomus argentipes*	*PaAbd2*	XP_059620308.1	28 September 2023
Blattodea	*Zootermopsis nevadensis*	*ZnAbd5*	XP_021921395.1	6 August 2017
Orthoptera	*Locusta migratoria*	*LmAbd5*	ASQ42725.1	7 August 2017
	*Locusta migratoria*	*LmAbd4*	ASQ42724.1	7 August 2017
	*Schistocerca gregaria*	*SgAbd5*	XP_049839296.1	10 August 2022
	*Schistocerca nitens*	*SnAbd5*	XP_049793153.1	5 August 2022
Phasmatoptera	*Timema douglasi*	*TdAbd2*	CAD7195650.1	24 November 2020
Hymenoptera	*Cataglyphis hispanica*	*ChAbd8*	XP_050448044.1	9 September 2022
	*Pseudomyrmex gracilis*	*PgAbd8*	XP_020289403.1	8 March 2017
	*Venturia canescens*	*VcAbd8*	XP_043269765.1	12 July 2024
	*Belonocnema kinseyi*	*BkAbd8*	XP_033208504.1	23 February 2022
Coleoptera	*Asbolus verrucosus*	*AvAbd5*	RZC34611.1	13 February 2019
	*Asbolus verrucosus*	*AvAbd2*	RZC40729.1	13 February 2019
	*Aromia moschata*	*AmAbd2*	KAJ8937326.1	13 February 2019
	*Anoplophora glabripennis*	*AgAbd8*	XP_018580236.2	10 January 2018
	*Aethina tumida*	*AtAbd8*	XP_019876049.1	11 August 2022
	*Acanthoscelides obtectus*	*AoAbd2*	CAH1983138.1	24 January 2023
	*Dalotia coriaria*	*DcAbd8*	XP_065163194.1	29 January 2025
	*Phaedon cochleariae*	*PcAbd2*	CAH1117680.1	18 October 2022
	*Nicrophorus vespilloides*	*NvAbd8*	XP_017774729.1	24 August 2016
	*Zophobas morio*	*ZmAbd5*	XP_063918219.198	27 March 2024
	*Tenebrio molitor*	*TmAbd5*	XP_068904910.1	8 October 2024
	*Tribolium castaneum*	*TcAbd5*	XP_970596.1	10 April 2024
	*Tribolium castaneum*	*TcAbd2*	XP_973729.2	10 April 2024
	*Tribolium castaneum*	*TcAbd8*	XP_969336.1	10 April 2024
	*Tribolium madens*	*TmadAbd5*	XP_044267494.1	16 October 2021

**Table 2:** Primer sequences used in the study.

Prime	Primer Sequences (5′-3′)	Application	Length (bp)
*TmAbd5*	F: GCGGAGAGTACACCTACAAGCTC	RT-qPCR	92
	R: GGTCTGTGTGTGGGGTTGGT		
*RPS3*	F: GTGGTCGTTTCTGGCAAACT		141
	R: CAACACTCCTTGCCTCAACA		
ds*TmAbd5*	F: taatacgactcactatagggAGTGGTGATCCTTCTCTGCG	dsRNA	176
	R: taatacgactcactatagggCGGCGTTCTTCAGCTCTC		
ds*GFP*	F: taatacgactcactatagggGTGGAGAGGGTGAAGG		571
	R: taatacgactcactatagggGGGCAGATTGTGTGGAC		

Lowercase letters indicate the T7 promoter sequence.

## Data Availability

The original contributions presented in this study are included in the article. Further inquiries can be directed to the corresponding author.
